# Urban-rural disparity in cancer mortality and changing trend in Tianjin, China, during 1999 and 2016

**DOI:** 10.1186/s12885-021-08907-0

**Published:** 2021-11-12

**Authors:** Wenlong Zheng, Hui Zhang, Dezheng Wang, Chong Wang, Shuang Zhang, Chengfeng Shen, Wei Li, Guohong Jiang

**Affiliations:** 1 NCDs Preventive Department, Tianjin Centers for Disease Control and Prevention, No. 6 Huayue Road, Hedong District, Tianjin, 300011 China; 2 School of Public Health, Tianjin Medical University, Tianjin, China

**Keywords:** Cancer, Mortality, Trend, Cancer transition

## Abstract

**Objective:**

Compare the urban-rural disparity in cancer mortality and changing trend during the past 18 years in Tianjin, China.

**Methods:**

Cancer death data were obtained from Tianjin All Cause of Death Registration System (CDRS), which covers the whole population of Tianjin. We calculated and compared the constituent ratio of cancer deaths, age-standardized mortality rate(ASR)and changing trends between urban and rural areas.

**Results:**

From 1999 to 2016, a total of 245,744 cancer deaths were reported, accounting 21.7% of all deaths in Tianjin. The ASR of total cancer mortality was higher in urban areas than in rural areas. A total of 33,739 persons were avoided dying of cancers in rural area compared to the urban death level from 1999 to 2016, which was 40.1% compare to the current level of rural areas. But the gap between urban and rural areas became narrowed gradually. The urban-rural ratios (urban/rural) of total cancer mortality changed from 1.76 (125.7/71.5)[95%CI,1.67,1.84] in 1999 to 1.11 (99.6/90.0)[95%CI,1.06,1.15] in 2016. The ASR of lung, liver and esophagus cancer became higher in rural areas than in urban areas in 2016.

**Conclusion:**

Cancer transition was obviously occurred in Tianjin and showed different speeds and big gap between urban and rural areas. Much more attention was needed to pay in rural areas which still have increasing trends in most cancers mortality recently.

## Introduction

According to the estimate from the World Health Organization (WHO) in 2015, cancer is the first or second leading cause of death before 70 years old in 91 of 172 countries [1]. In China, cancer became the leading cause of death since 2010 and an important issue affect public health [2]. Human development and urbanization have a closed affect on cancer’s incidence and mortality. Worldwide, cancers of the female breast, lung, colorectum, and prostate are the most common leading causes of cancer death in more developed countries, meanwhile, liver, stomach and esophagus cancer among males and cervical cancer among females are more popular in less developed countries [3, 4]. The ongoing displacement of infection-related and poverty-related cancers by those cancers that are already highly frequent in the most developed countries were observed in several studies at the country levels [4–7], but merely at city levels.

The gaps in work and lifestyles and the pace of economic development between urban and rural areas in China may have an impact on the level of cancer deaths and the types of epidemics. Comparative analysis of the urban and rural differences and changing trends of cancer death will have great significance for analyzing influencing factors of cancer death and developing relevant strategies.

Tianjin, located in the northeast of China, near the capital of Beijing, which have more than 10 million population, is a typical city of north China, and one of the four municipalities directly under the central government. We attempted to quantitatively compare the urban and rural differences of cancer mortality and changing trend in the past 17 years using the data in Tianjin CDRS.

## Methods

### Data sources

Cancer death data was obtained from Tianjin CDRS, which was established in 1984. Medical institutions at all levels in the city are required to report death cases through network direct report, and a three-level quality audit should be carried out by medical institutions, county CDC and municipal CDC step by step [8]. The information of tumor death cases and incidence cases is checked and supplemented mutually based on the International Cancer Registration Association, The Ninth Volume of Cancer Incidence on Five Continents and the Chinese Cancer Registration Guidance Manual [9]. The numbers of cancer deaths for all cancers combined (International Statistical Classification of Diseases and Related Health Problems-10th Revision codes C00-C97) and for 15 cancer types: esophagus (C15); stomach (C16); colorectum (C18-C21); liver (C22); gallbladder (C23-C24); pancreas (C25); lung (C33-C34, including trachea and bronchus); female breast (C50); cervix (C53); ovary (C56); prostate (C61); bladder (C67); brain, CNS (C70-C72); Hodgkin lymphoma (C81-C85, C88, C90, C96) and leukemia (C91-C95). Demographic data were obtained from Tianjin Public Security Bureau.

### Statistical analysis

Statistical analysis was performed to calculate the age-standardized mortality rate (ASR) using Segi’s world standard population age compositionof cancers in Tianjin. The trend of mortality, which include the average annual percentage change (AAPC) and 95% CI, were examined by Joinpoint Regression analysis (used Joinpoint Statistical Software, Version4.3). The AAPC was calculated as a geometrically weighted average of the various annual percent changes (APCs) from Joinpoint Regression analysis, with weights being equivalent to the length of each segment during the specified time interval [10]. The statistical significance of AAPC was ascertained comparing its magnitude with zero, and all in significant AAPCs were regarded as “not have significant changing trends”.

We calculated the theoretical avoided death number of cancers in rural areas compare to urban areas by the below formula. (*Ru*(*i*) − *Rr*(*i*)) is the difference value of cancers mortality rate between urban and rural areas in the year “i”. $\left(\frac{Ru(i)- Rr(i)}{Rr(i)}\right)$ is the percentage of the difference value to the rural mortality rate. So we use the rural cancer death number in the year “i”(*N*(*i*)) multiplied the percentage $\left(\frac{Ru(i)- Rr(i)}{Rr(i)}\right)$ get the theoretical avoided death number of cancers in the year “i”. Then $\sum \limits_{1999}^{2016}N(i)\times \left(\frac{Ru(i)- Rr(i)}{Rr(i)}\right)$ is the total theoretical avoided death number of cancers from 1999 to 2016.

1 $$ N=\sum \limits_{1999}^{2016}N(i)\times \left(\frac{Ru(i)- Rr(i)}{Rr(i)}\right) $$

Where:

*N* was the total theoretical avoided death number of cancers in rural areas compare to urban areas from 1999 to 2016.

*N(i)* was the cancers death number of rural areas in the year of i.

*Ru(i)* was the ASR of urban areas.

*Rr(i)* was the ASR of rural areas.

We calculated the percentage of the theoretical avoided death number of cancers to the current death number of cancers in rural areas from 1999 to 2016 by the below formula:

2 $$ R=\left(\frac{N}{Nd}\right)\times 100\% $$

“*R*” is the percentage of the theoretical avoided death number of cancers to the current death number of cancers in rural areas from 1999 to 2016.

*N* was the total theoretical avoided death number of cancers in rural areas compare to urban areas from 1999 to 2016.

*Nd* was the real death number of cancers in rural areas from 1999 to 2016.

## Result

### Constituent ratio of cancer deaths in urban and rural areas

From 1999 to 2016, a total of 164,237,762 person years were observed in Tianjin in the study, with 82,669,481(50.3%) males, 81,568,281(49.7%) females, 83,529,479(50.9%) in urban areas, 80,708,283 (49.1%) in rural areas. A total of 245,744 cancer deaths were reported during 1999 to 2016, accounting for 21.7% (245,744/1131117) of all deaths in Tianjin, and these included 142,227 (57.9%) males, 103,517 (42.1%) females, and 161,558 (65.7%) in urban areas, 84,186 (34.3%) in rural areas.

**Fig. 1:**
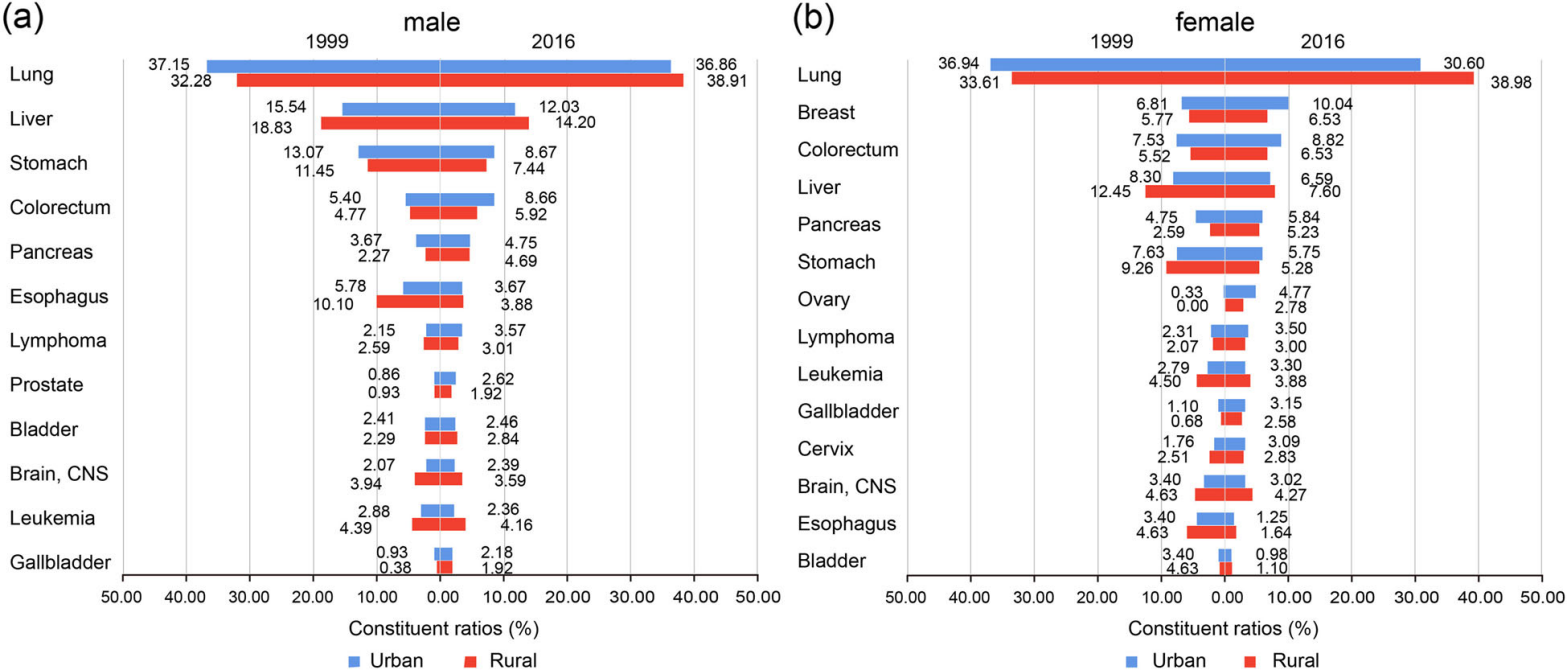
The male’s (**a**) and female’s (**b**) Cancer constituent ratios in the year 1999 and 2016 in Urban and Rural areas of Tianjin, China

In 1999, the top 5 cancer deaths for male were cancers of lung, liver, stomach, esophagus and colorectum both in urban and rural area, accounting for 76.97 and 77.43% separated. They changed to lung, liver, stomach, colorectum and pancreas in 2016, both in urban and rural area, accounting for 70.97 and 71.16% respectively. (Fig. 1.A).

For female, the top 5 cancer deaths were quite different between urban and rural areas. In 1999, they were cancers of lung, liver, stomach, colorectum and breast in urban area, accounting for 59.68% cancer deaths, comparing to lung, liver, stomach, esophagus and breast in rural area, accounting for 67.22% cancer deaths. (Fig. 1.B) In 2016, the order changed to lung, breast, colorectum, liver and pancreas in urban area, accounting for 61.89% cancer deaths. But lung, liver, breast, colorectum and stomach cancer were the top 5 cancers in rural area in 2016, accounting for 64.92% cancer deaths. (Fig. 1.B).

### Cancer mortalities differences between urban and rural areas from 1999 to 2016

The ASR of total cancer mortality in urban areas was higher than that in rural areas, with ratio 1.76 (125.7/71.5) [95%CI,1.67,1.84] in 1999 and 1.11 (99.6/90.0)[95%CI,1.06,1.15] in 2016. A total of 33,739 persons were avoided dying from cancers in rural area compare to the urban death level from 1999 to 2016, which was 40.1% compare to the current level of rural areas (male 42.6%, female 36.5%). The death avoided percentages have a big variation among different cancers. From high to low were ovary (199.8%), prostate (119.0%), gallbladder (114.8), colorectum (85.3%), breast (66.7%), pancreas (59.6), lymphoma (54.1%), stomach (50.7%), bladder (40.2%), lung (39.5%), esophagus (16.2%), cervix (19.3), liver (11.1%), brain and CNS (4.9%) and leukemia (− 9.4%). But the gap between urban and rural areas gradually narrowed. The urban-rural ratios (urban/rural) of lung, live, stomach, esophagus, colorectum and pancreas were 1.98, 1.37, 1.80, 1.07, 2.19 and 3.06 in 1999, changing to 0.98, 0.95 1.27, 1.00, 1.55, 1.18 in 2016. The urban-rural ratios became less in 2016 of 14 kinds of cancers included in the statistics except prostate cancer. (Table 1).

**Table 1:** The differences of cancer mortality rates (ASR) between urban and rural areas in Tianjin, China

type	Urban(1/100000)		Rural(1/100000)		1999	2016	N^a^	R^b^(%)
	1999	2016	1999	2016	(U/R)	(U/R)	(person)	
total	125.7	99.6	71.5	90.0	1.76	1.11	33,739	40.1
male	151.6	119.1	91.1	107.5	1.66	1.11	20,770	42.6
female	99.8	80.0	57.6	73.4	1.73	1.09	12,969	36.5
lung	46.6	34.2	23.5	35.0	1.98	0.98	12,505	39.5
liver	15.9	9.8	11.6	10.3	1.37	0.95	1404	11.1
stomach	13.7	7.5	7.6	5.9	1.80	1.27	3513	50.7
esophagus	6.5	2.7	6.1	2.7	1.07	1.00	572	16.2
colorectum	7.9	8.7	3.6	5.6	2.19	1.55	3863	85.3
pancreas	5.2	5.2	1.7	4.4	3.06	1.18	1943	59.6
leukemia	3.6	2.7	3.2	3.6	1.13	0.75	− 298	−9.4
brain and CNS	3.3	2.6	3.0	3.5	1.10	0.74	150	4.9
breast (female)	6.8	8.0	3.3	4.8	2.06	1.67	1583	66.7
lymphoma	2.8	3.5	1.7	2.7	1.65	1.30	1102	54.1
bladder	2.3	1.9	1.2	1.9	1.92	1.00	444	40.2
gallbladder	1.3	2.9	0.4	2.0	3.25	1.45	1624	114.8
Prostate	1.3	3.1	0.8	1.9	1.63	1.63	2487	119.0
Cervix	1.8	2.5	1.4	2.1	1.29	1.19	372	19.3
ovary	0.3	3.8	0.0	2.1	–	1.81	4974	199.8

a“N” is the avoided death number of cancers in rural areas compare to urban death level

b“R” is the percentage of the avoided death number to the current death number of rural areas from 1999 to 2016

“U/R” = age standard mortality rate of urban areas/ age standard mortality rate of rural areas

### Changing trends of cancer mortalities in urban and rural areas from 1999 to 2016

For urban areas from 1999 to 2016, the ASRs of cancer mortality in whole population (AAPC = − 1.2%, [− 1.7, − 0.7%]), males (AAPC = − 1.1%, [− 1.6, − 0.7%]) and females (AAPC = − 1.3%, [− 1.8, − 0.8%]) all have significant decreasing trends. (Table 2) But they all have increasing trends in rural areas, with AAPC of 1.1% [0.1, 2.1%] (total), 1.0% [0.0,1.9%] (male) and 1.6% [1.1, 2.1%] (female) respectively. (Table 3).

**Table 2:** The changing trend of cancers mortality from 1999 to 2016 in urban area of Tianjin, China

type	AAPC(%)	trend 1		trend 2	
		years	APC(%)	years	APC(%)
total	− 1.20^a^(−1.7,-0.7)	1999–2016	− 1.20^a^(− 1.7,-0.7)		
male	− 1.13^a^(− 1.6, -0.7)	1999–2016	−1.13^a^(− 1.6, -0.7)		
female	−1.31^a^(− 1.8, -0.8)	1999–2016	− 1.31^a^(− 1.8, -0.8)		
lung	− 1.43^a^(− 2.0, -0.9)	1999–2016	− 1.43^a^(− 2.0, -0.9)		
liver	− 2.49^a^(− 3.0, −2.0)	1999–2016	-2.49^a^(− 3.0, -2.0)		
stomach	−3.15^a^(− 3.7, -2.6)	1999–2016	−3.15^a^(− 3.7, -2.6)		
esophagus	−5.02^a^(− 5.6, -4.4)	1999–2016	−5.02^a^(− 5.6, -4.4)		
colorectum	0.82^a^(0.3,1.3)	1999–2016	0.82^a^(0.3,1.3)		
pancreas	0.27(− 0.5,1.1)	1999–2016	0.27(− 0.5,1.1)		
leukemia	−0.93^a^(−1.7,− 0.2)	1999–2016	-0.93^a^(− 1.7,-0.2)		
brain and CNS	0.17(− 1.2,1.6)	1999–2016	0.17(− 1.2,1.6)		
breast (female)	− 0.01(− 0.8,0.8)	1999–2016	− 0.01(− 0.8,0.8)		
lymphoma	1.58^a^(0.7,2.5)	1999–2016	1.58^a^(0.7,2.5)		
bladder	−2.52^a^(−3.6,-1.4)	1999–2016	− 2.52^a^(− 3.6,-1.4)		
gallbladder	2.11^a^(0.5,3.7)	1999–2016	2.11^a^(0.5,3.7)		
Prostate	4.22^a^(2.7,5.4)	1999–2016	4.22^a^(2.7,5.4)		
Cervix	2.3(−0.3,5.1)	1999–2010	−3.37^a^(−6.1,-0.6)	2010–2016	13.65^a^(6.6,21.2)
ovary	15.1^a^(8.6,22.0)	1999–2003	71.75^a^(31.9123.6)	2003–2016	1.75(−0.5,4.0)

^a^The AAPC or ACP has significantly different from zero (*P* < .05)

**Table 3:** The changing trend of cancers mortality from 1999 to 2016 in rural area of Tianjin, China

type	AAPC	trend 1		trend 2		trend3	
		years	APC	years	APC	years	APC
total	1.10^a^(0.1,2.1)	1999–2004	−0.99(−4.1,2.2)	2004–2016	1.99^a^(1.2,2.8)		
male	1.00^a^(0.0,1.9)	1999–2005	−1.06(−3.4,1.4)	2005–2016	2.10^a^(1.2,3.1)		
female	1.57^a^(1.1,2.1)	1999–2016	1.57^a^(1.1,2.1)				
lung	2.47^a^(1.8,3.1)	1999–2016	2.47^a^(1.8,3.1)				
liver	−1.44^a^(−2.2, -0.7)	1999–2016	− 1.44^a^(− 2.2, -0.7)				
stomach	−1.46^a^(−2.0, -0.9)	1999–2016	− 1.46^a^(− 2.0, -0.9)				
esophagus	−4.40^a^(−6.1, -2.7)	1999–2005	−9.88^a^(−13.5, -6.2)	2005–2016	− 1.28(−3.2,0.7)		
colorectum	2.40^a^(1.1,3.6)	1999–2004	−1.20(−4.9,2.7)	2004–2016	3.87^a^(2.9,4.8)		
pancreas	4.73^a^(3.8,5.6)	1999–2016	4.73^a^(3.8,5.6)				
leukemia	0.22(−0.5,0.9)	1999–2016	0.22(−0.5,0.9)				
brain and CNS	0.90^a^(−1.8,3.6)	1999–2003	−7.68(− 17.4,3.1)	2003–2016	3.74^a^(1.9,5.5)		
breast (female)	2.76^a^(1.6,3.9)	1999–2016	2.76^a^(1.6,3.9)				
lymphoma	2.00(−1.6,5.8)	1999–2005	−8.78(− 14.7,-2.4)	2005–2012	13.43^a^(6.7,20.6)	2012–2016	0.25(−8.6,10.0)
bladder	0.71(−1.3,2.8)	1999–2016	0.71(− 1.3,2.8)				
gallbladder	8.05^a^(6.3,9.8)	1999–2016	8.05^a^(6.3,9.8)				
Prostate	6.45^a^(4.1,8.9)	1999–2016	6.45^a^(4.1,8.9)				
Cervix	3.1(−0.9,7.2)	1999–2008	−4.2(−10.0,1.9)	2008–2016	11.93^a^(5.4,18.9)		
ovary	10.7^a^(7.2,14.4)	1999–2016	10.7^a^(7.2,14.4)				

^a^The AAPC or ACP has significantly different from zero (*P* < .05)

**Fig. 2:**
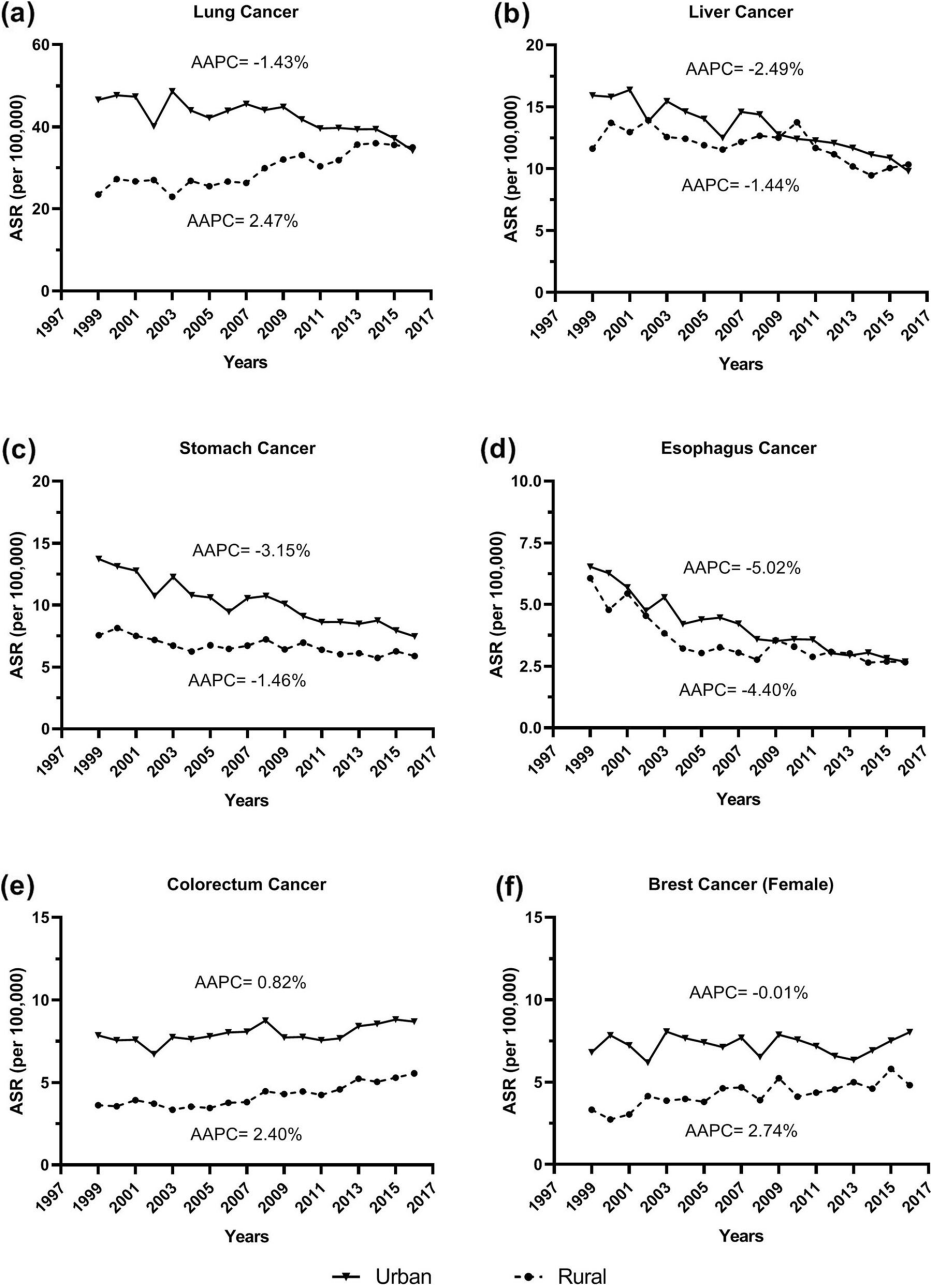
The trends of major cancers’ age-standardized mortality rate (ASR) from 1999 to 2016 in Tianjin, China

Of the top 5 cancer deaths, there were 4 having decreasing trends in urban areas, including lung (AAPC = -1.4%, [− 2.0,-0.9%]), liver (AAPC = − 2.5%, [− 3.0, − 2.0%]), stomach (AAPC = − 3.2%, [− 3.7, − 2.6%]) and esophagus (AAPC = − 5.0%, [− 5.6, − 4.4%]).(Table 2) But the lung cancer in rural areas had quick increasing trend (AAPC = 2.5%, [1.8, 3.1%]). (Table 3) The ASR of lung cancer, liver cancer and esophagus cancer in rural areas became higher than that in urban areas in 2016. (Fig. 2) The ASRs of colorectum, prostate, cervix and ovary cancer all have increasing trends both in urban and rural areas. (Tables 2, 3).

## Discussion

The prevalent pattern of cancer was closely related to human development. There are two features [2–7]. The first is the different constituent ratio of death. Cancers of lung, colorectum, prostate and female breast are the most common leading causes of cancer death in more developed countries, which are often ascribed to a so-called westernization lifestyle. And Liver, stomach and cervical cancer among females, called infection-related and poverty-related cancers, are more popular in less developed countries [4]. The second feature is high-income countries have higher cancer death rate and decreasing trend, but low-income countries just on the contrary, have lower cancer death rate and increasing trend [11]. The results in our study showed that the cancer prevalent pattern in 2016 was more closed to high developed countries than that in 1999. Moreover, the prevalent pattern and trend of cancer in urban areas were more closed to high developed countries, and those in rural areas were more closed to middle developed countries. The analysis of cancer species showed that there was a high consistency between the cancer species with a large gap between urban and rural areas and the cancer species with a rapid growth, such as ovary, prostate, gallbladder, colorectum, breast, pancreas. They are also the main cancer species in developed countries [1, 3, 12, 13]. These have strengthened the evidence of the relation between cancer pattern and human development because it can exclude some confounding factors such as dietary habit among different countries.

The phenomenon of cancer transitions was obviously occurred in Tianjin from 1999 to 2016, especially for women’s, whose top 3 cancers death have changed from lung, liver, stomach to lung, breast, colorectum. Analyze cancer transitions has great significant to conform priorities of controlling strategies. From 1999 to 2016, lung cancer has always been the leading cause of cancer deaths in Tianjin both in urban and rural areas, accounting for more than a third of cancer deaths. That was mainly because of high smoking rate both in urban and rural areas. Men born before 1980 in Tianjin had high smoking rates (more than 58%) [14]. After the 1980 generation, both men’s and women’s smoking rates in urban areas had great decline, but still keep high in rural men [14]. Accordingly, we can see declining trend of lung cancer mortality in urban areas and increasing trend in rural areas. Tianjin implemented Smoke-free Regulation in 2012, and the acute myocardial infarction (AMI) decreased significantly [15]. However, its effect on lung cancer mortality is not as yet evident. Much more work was needed on tobacco control, especially for rural men.

In the past 18 years, the death rates of cancers of esophagus, stomach, liver have declined to a large extent, which could closely relate to infection control and nutritional improvement [16–18]. 75% of liver cancer in China is attributable to modifiable risk factors, including hepatitis B infection, aflatox in contamination, smoking, excessive alcohol consumption, etc. [19] The control of hepatitis B infection is an important reason for the decline of liver cancer mortality in China [20]. In 2010, the reported incidence of hepatitis B in the population under 20 years old in Tianjin decreased by 33.00% compared with that in 1992 [21], and the incidence of acute hepatitis B in 2013 decreased by 80.06% compared with that in 2005 [22]. The decrease of stomach cancer and esophagus cancer is mainly related to the improvement of nutrition, and the improvement of screening and treatment [18]. But China still have the maximal incidence and death numbers of these digestive system cancers [1]. Esophageal squamous-cell carcinoma is the main type in China [23]. A meta study showed that adequate intake of fresh fruits and vegetables can reduce esophageal squamous-cell carcinoma incidence rate significantly [ 24].In 2005, 85,421 cases of esophageal cancer deaths in the Chinese population were attributed to smoking, drinking alcohol, and insufficient intake of vegetables and fruits, accounting for about 46% of esophageal cancer deaths [25]. The contribution of insufficient fruit intake to esophageal cancer death was 27.1% in men and 28.0% in women [25]. The median of average daily consumption of fruit in Chinese people was only 6.4 g in 2015 [26], still far from the recommended intake.

Our study showed the mortality rate of the sedigestive system cancers in rural areas became closer to or exceed that in urban areas. Recent surveys also showed that the smoking rate and drinking rate in young age groups in rural areas were higher than these in urban areas in Tianjin city [14, 27]. Take measures to reduce the hepatitis B infection, aflatox in contamination, smoking, excessive alcohol consumption to a lower level, and raise fruits intake are important strategies, especially for rural areas.

On the other hand, more attention was needed to pay to the increase mortality among colorectum, breast, pancreas, prostate and ovary cancers, both in urban and rural areas. While each of these cancers has its own contributing factors, rising rates of obesity and a decline in physical activity may be a major contributing together [28–34]. The rate of overweight and obesity (BMI ≥ 24) among Chinese adults increased from 25.1% in 1997 to 46.5% in 2013 [35]. From 1990 to 2013, the rate of death caused by high BMI increased rapidly in China, which was attributed to the large increase in deaths from pancreatic cancer, colorectum cancer and breast cancer [36]. The overweight rate of adults in Tianjin is 31.9%, and the obesity rate is 14.2% [37], which is one of the most serious overweight and obesity regions in China. In addition, the improvement of diagnosis techniques and the development of early screening programs are also contributing to the increase in morbidity and mortality of cancers. Such as gradual implementation of prostate-specific antigen screening and improved biopsy techniques [38], cervical and breast cancer screening program in rural women from 2009 [39].

### Limitations

Our analysis has several limitations. Firstly, the different levels in death diagnosis between urban and rural areas could affect the analysis results. Although there were three-level quality audit, the differences in diagnosis of death between urban and rural areas cannot be completely avoided. Secondly, for the difference between urban and rural areas, we can only analyze the possible causes based on the previous correlation studies of risk factors. There is no relevant data for quantitative analysis of influencing factors and their respective proportions. Beside these well known risk factors, such as life-style, environment, medical condition, population mobility is also an important factors that could affect the results. Much more work was needed to be done to analyse the difference.

## Conclusions

We found great difference between urban and rural areas both in prevalent pattern of cancer and also the trend. It brings us a new subject of research into the causes of this urban-rural gap for control strategies. Much more attention was needed to rural areas which still have increasing trends in most cancers’ mortality.

## Data Availability

The datasets used and/or analysed during the current study available from the corresponding author on reasonable request.
